# Supplementary material for: Marine outfall discharges contribute to coastal microplastic pollution and the spread of antimicrobial resistance
Source: PLoS One. 2025 Aug 19;20(8):e0329599. doi: 10.1371/journal.pone.0329599 (PMC12364349; doi:10.1371/journal.pone.0329599)
Supplement: S1 Table — The different antibiotics used in this study with the growth inhibition zone standards for Enterobacteriaceae. (DOCX) [file pone.0329599.s001.docx]

**Marine Outfall Discharges Contribute to Coastal Microplastic Pollution and the Spread of Antimicrobial Resistance**

# **Supporting information**

**S1 Table 1:** The different antibiotics used in this study with the growth inhibition zone standards for Enterobacteriaceae.

| **Antibiotics** | **Disk content** | **Interpretive Categories and Zone Diameter Breakpoints, nearest whole mm** | | |
| --- | --- | --- | --- | --- |
|  |  | **S** | **I** | **R** |
| **Class 1: Penicillin** | | | | |
| Ampicillin | 10 µg | ≥ 17 | 14–16 | ≤ 13 |
| **Class 2: B - lactam combination agents** | | | | |
| Amoxicillin- clavulanate | 20 / 10 µg | ≥ 18 | 14 – 17 | ≤ 13 |
| **Class 3: Carbapenems** | | | | |
| Doripenem | 10 µg | ≥ 23 | 20 – 22 | ≤ 19 |
| Ertapenem | 10 µg | ≥ 22 | 19 - 21 | ≤ 18 |
| Imipenem | 10 µg | ≥ 23 | 20 – 22 | ≤ 19 |
| Meropenem | 10 µg | ≥ 23 | 20 – 22 | ≤ 19 |
| **Class 4: Aminoglycosides** | | | | |
| Gentamycin | 10 µg | ≥ 15 | 13 - 14 | ≤ 12 |
| Kanamycin | 30 µg | ≥ 18 | 14 – 17 | ≤ 13 |
| Streptomycin | 10 µg | ≥ 15 | 12 - 14 | ≤ 11 |
| **Class 5: Macrolides** | | | | |
| Azithromycin | 15 µg | ≥ 13 | - | ≤ 12 |
| **Class 6: Tetracycline** | | | | |
| Tetracycline | 30 µg | ≥ 15 | 12 – 14 | ≤ 11 |
| **Class 7: Quinolones and fluoroquinolones** | | | | |
| Ciprofloxacin | 5 µg | ≥ 21 | 16 - 20 | ≤ 15 |
| Nalidixic acid | 30 µg | ≥ 19 | 14 - 18 | ≤ 13 |
| **Class 8: Folate pathway antagonists** | | | | |
| Trimethoprim-sulfamethoxazole | 1.25 / 23.75 µg | ≥ 16 | 11 - 15 | ≤ 10 |
| **Class 9: Phenicols** | | | | |
| Chloramphenicol | 30 µg | ≥ 18 | 13 - 17 | ≤ 12 |
| **Class 10: Fosfomycin** | | | | |
| Fosfomycin | 200 µg | ≥ 16 | 13 - 15 | ≤ 12 |
