# Supplementary material for: Marine outfall discharges contribute to coastal microplastic pollution and the spread of antimicrobial resistance
Source: PLoS One. 2025 Aug 19;20(8):e0329599. doi: 10.1371/journal.pone.0329599 (PMC12364349; doi:10.1371/journal.pone.0329599)
Supplement: S2 Table — (DOCX) [file pone.0329599.s002.docx]

**Marine Outfall Discharges Contribute to Coastal Microplastic Pollution and the Spread of Antimicrobial Resistance**

**S1 Table 2:** Accession numbers obtained from GenBank

| **Accession** | **Sequence ID** |
| --- | --- |
| OR178026 | Citrobacter_sp._R351E |
| OR178027 | Citrobacter_sp._R441E |
| OR178028 | Citrobacter_sp._R521E |
| OR178029 | Citrobacter_sp._R561E |
| OR178030 | Enterobacter_sp._R211E |
| OR178031 | Enterobacter_sp._R241E |
| OR178032 | Enterobacter_sp._R313E |
| OR178033 | Enterobacter_sp._R471E |
| OR178034 | Enterobacter_sp._R61i |
| OR178035 | Klebsiella_sp._R761E |
| OR178036 | Escherichia_sp._R641E |
| OR178037 | Escherichia_sp._R652E |
| OR178038 | Escherichia_sp._R732E |
| OR178039 | Klebsiella_sp._R12i |
| OR178040 | Klebsiella_sp._R212E |
| OR178041 | Klebsiella_sp._R251E |
| OR178042 | Klebsiella_sp._R25i |
| OR178043 | Klebsiella_sp._R341E |
| OR178044 | Klebsiella_sp._R342E |
| OR178045 | Klebsiella_sp._R45i |
| OR178046 | Klebsiella_sp._R63i |
| OR178047 | Klebsiella_sp._R762E |
| OR178048 | Pseudomonas_sp._R213E |
| OR178049 | Pseudomonas_sp._R253E |
| OR178050 | Pseudomonas_sp._R273E |
| OR178051 | Pseudomonas_sp._R423E |
| OR178052 | Pseudomonas_sp._R433E |
| OR178053 | Pseudomonas_sp._R463E |
| OR178054 | Pseudomonas_sp._R731E |
| OR178055 | Vibrio_sp._R512E |
