# Supplementary material for: Marine outfall discharges contribute to coastal microplastic pollution and the spread of antimicrobial resistance
Source: PLoS One. 2025 Aug 19;20(8):e0329599. doi: 10.1371/journal.pone.0329599 (PMC12364349; doi:10.1371/journal.pone.0329599)
Supplement: Attachment — Submitted filename: Response to Reviewers.docx [file pone.0329599.s003.docx]

**Addressing the Insufficiency of Marine Outfall Regulations in Mitigating Microplastic and AMR Pollution from Wastewater Treatment**

*R Bhikhoo^1^, K Bezuidenhout^2^, L.G Molale-Tom^1^, C Mienie^1^, CC Bezuidenhout^1^*

*^1^Unit for Environmental Sciences and Management: Microbiology, North-West University, South Africa*

*^2^South African Research Chair: Cities, Law and Environmental Sustainability (CLES)*

*North-West University, South Africa*

1. *Please ensure that your manuscript meets PLOS ONE's style requirements, including those for file naming.*

Addressed.

2. *In your Methods section, please provide additional information regarding the permits you obtained for the work. Please ensure you have included the full name of the authority that approved the field site access and, if no permits were required, a brief statement explaining why.*

**Ethical and Legal Compliance**

No specific permits were required for wastewater sampling at the outfall site, as this study did not involve protected areas, endangered species, or private land access. The sampling was conducted in accordance with South African environmental guidelines for non-invasive marine research. All field personnel completed institutional indemnity forms acknowledging safety protocols and legal compliance. The Municipality’s Environmental Management Department was notified of the sampling activities as a courtesy measure, and they have sent out officials to assist.

The permits mentioned in the methodology and results are required by municipalities as part of the South African legislation when wastewater is discharged into waterways.

3. *Thank you for stating the following financial disclosure: This work is based on the research supported in part by the National Research Foundation of South Africa (Grant Numbers: UID:118755, UID:115581 and UID:150936). The views expressed are those of the authors and not of the funding agency.*

*Please state what role the funders took in the study. If the funders had no role, please state: "The funders had no role in study design, data collection and analysis, decision to publish, or preparation of the manuscript."*

*If this statement is not correct you must amend it as needed. Please include this amended Role of Funder statement in your cover letter; we will change the online submission form on your behalf.*

The funders had no role in study design, data collection and analysis, decision to publish, or preparation of the manuscript.

4. *Thank you for stating the following in the Acknowledgments Section of your manuscript: This work is based on the research supported in part by the National Research Foundation of South Africa (Grant Numbers: UID:118755, UID:115581 and UID:150936). The views expressed are those of the authors and not of the funding agency. We note that you have provided funding information that is not currently declared in your Funding Statement. However, funding information should not appear in the Acknowledgments section or other areas of your manuscript. We will only publish funding information present in the Funding Statement section of the online submission form.*

*Please remove any funding-related text from the manuscript and let us know how you would like to update your Funding Statement. Currently, your Funding Statement reads as follows: This work is based on the research supported in part by the National Research Foundation of South Africa (Grant Numbers: UID:118755, UID:115581 and UID:150936). The views expressed are those of the authors and not of the funding agency.*

Please add the following to the funding statement: “The funders had no role in study design, data collection and analysis, decision to publish, or preparation of the manuscript”.

5. *Thank you for stating the following in the Competing Interests section:*

*Raeesa Bhikhoo performed the Microbiology work under the supervision of Cornelius Carlos Bezuidenhout, Charlotte Mienie and Lesego Gertrude Molale-Tom. Krisdan Bezuidenhout contributed the regulatory framework contents.*

*All authors contributed equally to the manuscript and approved the final version submitted. Please confirm that this does not alter your adherence to all PLOS ONE policies on sharing data and materials, by including the following statement: ""This does not alter our adherence to PLOS ONE policies on sharing data and materials.” (as detailed online in our guide for authors http://journals.plos.org/plosone/s/competing-interests). If there are restrictions on sharing of data and/or materials, please state these. Please note that we cannot proceed with consideration of your article until this information has been declared. Please include your updated Competing Interests statement in your cover letter; we will change the online submission form on your behalf.*

This does not alter our adherence to PLOS ONE policies on sharing data and materials.

This information will be added to the cover letter.

6. *Your ethics statement should only appear in the Methods section of your manuscript. If your ethics statement is written in any section besides the Methods, please move it to the Methods section and delete it from any other section. Please ensure that your ethics statement is included in your manuscript, as the ethics statement entered into the online submission form will not be published alongside your manuscript.*

The ethics statement has been added to the methodology under: “Ethical and Legal Compliance for sampling wastewater and approval of study”

7. *We note that Figure 1 in your submission contain [map/satellite] images which may be copyrighted. All PLOS content is published under the Creative Commons Attribution License (CC BY 4.0), which means that the manuscript, images, and Supporting Information files will be freely available online, and any third party is permitted to access, download, copy, distribute, and use these materials in any way, even commercially, with proper attribution. For these reasons, we cannot publish previously copyrighted maps or satellite images created using proprietary data, such as Google software (Google Maps, Street View, and Earth). For more information, see our copyright guidelines:* [*http://journals.plos.org/plosone/s/licenses-and-copyright*](http://journals.plos.org/plosone/s/licenses-and-copyright)*.*

**Figure 1:** Effluent discharge sites along the South African coast. Map adapted by Lohan Bredenhann in ArcGIS Desktop 10.7 using open-source coastline data (Natural Earth) and outfall locations compiled from the Department of Environmental Affairs’ (DEA) 2013–2014 South African National Magisterial Dataset. This work replaces a reference map from Polley, (2015) for compliance with CC BY 4.0 licensing.

The map was adapted by Lohan Bredenhann in ArcGIS Desktop 10.7 and open-access coastline data (Natural Earth). Effluent outfall locations were georeferenced from the Department of Environmental Affairs’ (DEA) 2013–2014 South African National Magisterial Dataset. This original work replaces a copyrighted reference map from Polley, (2015) to comply with PLOS licensing requirements. The ArcGIS map was independently adapted by Lohan Bredenhann to avoid copyright restrictions associated with the original reference map. Data sources are cited in the figure caption.

- Department of Environmental Affairs (DEA) (2013-2014) South African National Magisterial Dataset. Pretoria: Government of South Africa.
- The Environmental Systems Research Institute's (ESRI) software, ArcGIS Desktop 10.7, was used to make the map.
- Polley, M. (2015) ‘Waste water “threat” to Cape Town’s beaches: Photographs fuel fear of contaminated ocean along “platinum mile”’, Weekend Argus, 13 June.

8. *Below mentioned are the query (marked ?) with respect to the text of the manuscript. Clarify Each one separately.*
   a. ***The wastewater then undergoes a tertiary phase, whereby disinfection occurs that aids the removal of pathogenic microorganisms.***

***How do these pathogenic microorganisms get removed in tertiary treatment, and what about the non-pathogenic microorganisms?***

In South Africa, tertiary wastewater treatment is primarily aimed at eliminating pathogens and nutrients such as phosphorus and nitrogen to mitigate the risk of eutrophication in water bodies. Chlorine is the most common disinfectant used across the country because it effectively inactivates bacteria, viruses, and protozoan cysts by damaging their cellular structures. The treatment processes that target pathogenic organisms also reduce non-pathogenic microorganisms as a secondary outcome of the treatment processes (DWAF, 2004; Momba et al., 2006).

- DWAF (Department of Water Affairs and Forestry). (2004). South African water quality guidelines: Volume 1: Domestic water use. 2nd ed. Pretoria: DWAF.
- Momba, M.N.B., Kfir, R., Venter, S.N. and Cloete, T.E. (2006). ‘An overview of biofilm formation in distribution systems and its impact on the deterioration of water quality’, Water SA, 32(1), pp. 1–10.
  b. ***South Africa is reported to have fourteen marine sewage plant outfalls, as reflected in Figure 1. Define marine sewage plant outfalls?***

A marine sewage outfall is a pipeline that releases treated or partially treated wastewater into the ocean. These outfalls typically release effluent underwater at significant depths, to facilitate natural dilution and dispersion of pollutants, including pathogens and organic matter. Wastewater undergoes minimal treatment prior to discharge, relying on the ocean's natural processes to further reduce pathogen levels and pollutant concentrations. (EPA, 2021; WHO, 2017).

- EPA (Environmental Protection Agency). (2021). National pollutant discharge elimination system (NPDES): Marine sewage outfalls. Available at: https://www.epa.gov (Accessed: 15 October 2023).
- WHO (World Health Organization). (2017). Guidelines for safe recreational water environments: Volume 1 – Coastal and fresh waters. Geneva: WHO Press.
  c. ***According to the latest Green Drop report, a comprehensive audit of 850 wastewater systems revealed concerning findings: 208 plants were classified as being at critical risk, while 250 were deemed to be at high risk. On what basis/criteria were these 208 plants classified as being at critical risk, while 250 were deemed to be at high risk?***

In South Africa, wastewater treatment plants (WWTPs) are classified using a comprehensive risk assessment framework. Plants are categorized as either critical risk or high risk based on their Cumulative Risk Rating (CRR), which evaluates factors including design capacity, operational inflow, technical skills, and final effluent quality. Assessment also covers key performance areas such as process control, maintenance practices, and compliance with wastewater quality standards. WWTPs experiencing significant operational challenges, poor effluent quality, or inadequate management practices typically receive critical risk classification, while those with moderate issues are designated as high risk. This system helps identify facilities needing urgent intervention to protect environmental and public health (DWS, 2018, Marnus *et al.,* 2010).

- DWS (Department of Water and Sanitation). (2018). Green Drop Certification Programme: Risk rating methodology for wastewater treatment works. Pretoria: DWS.
- Manus, L. and van der Merwe-Botha, M., 2010. Raising wastewater treatment performance through incentive and risk-based targeted regulation. Proceedings, Water Institute of South Africa (WISA) 2010.
  d. ***Sea water contains many persistent organic pollutants, thus plastic debris in the ocean acts as a cleaning agent by absorbing these pollutants onto the surface of plastics or MPs. It is a contradictory statement as microplastics are also the cause of pollution.***

While microplastics have the capacity to absorb and concentrate persistent organic pollutants (POPs), this process does not eliminate these pollutants from the ocean; instead, it redistributes them within the marine environment. When marine organisms ingest microplastics, the associated pollutants can bioaccumulate and bio-magnify through the food chain, posing risks to marine life and human health. Additionally, microplastics themselves act as a significant source of pollution, introducing harmful chemicals into marine ecosystems and causing physical harm through ingestion and entanglement. As highly persistent pollutants, microplastics worsen environmental challenges rather than alleviating them, solidifying their role as a major contributor to marine pollution rather than it being a solution (Andrady, 2011; Rochman et al., 2016; UNEP, 2021).

- Andrady, A.L. (2011). ‘Microplastics in the marine environment’, Marine Pollution Bulletin, 62(8), pp. 1596–1605.
- Rochman, C.M., Browne, M.A., Halpern, B.S., Hentschel, B.T., Hoh, E., Karapanagioti, H.K., Rios-Mendoza, L.M., Takada, H., Teh, S. and Thompson, R.C. (2016). ‘Classify plastic waste as hazardous’, Nature, 494(7436), pp. 169–171.
- UNEP (United Nations Environment Programme). (2021). Marine plastic pollution: Global lessons and research to inspire action and guide policy change. Nairobi: UNEP.
  e. ***Due to the particulate size of MPs, it is made available for ingestion by marine organisms ranging from zooplankton to mammals who mistake it for food. Define the size of MPs?***

Microplastics (MPs) are defined as plastic particles measuring less than 5 millimeters (mm) in diameter. This includes both primary microplastics, which are intentionally produced at small sizes, and secondary microplastics, which are formed through the fragmentation and degradation of larger plastic debris. Plastic particles that are under 1 mm to 10 µm are also categorized as microplastics. They are small in size and resemble food particles allowing ingestion by marine organisms (GESAMP, 2015; Wright et al., 2013; Thompson et al., 2009).

- GESAMP (Joint Group of Experts on the Scientific Aspects of Marine Environmental Protection). (2015). Sources, fate and effects of microplastics in the marine environment: A global assessment. London: IMO/FAO/UNESCO-IOC/UNIDO/WMO/IAEA/UN/UNEP/UNDP.
- Thompson, R.C., Moore, C.J., vom Saal, F.S. and Swan, S.H. (2009). ‘Plastics, the environment and human health: Current consensus and future trends’, Philosophical Transactions of the Royal Society B: Biological Sciences, 364(1526), pp. 2153–2166.
- Wright, S.L., Thompson, R.C. and Galloway, T.S. (2013). ‘The physical impacts of microplastics on marine organisms: A review’, Environmental Pollution, 178, pp. 483–492.
  f. ***Influent and effluent samples were taken by using the dip-sample technique and plastic pieces were then obtained by using mesh sieves in sizes of 20 μm, 100 μm, and 200 μm with a diameter of the sieve being 200 mm each (Filtration group, South Africa). What is the rationale behind taking these sizes as MPs can be smaller than this.***

The selection of 20 μm, 100 μm, and 200 μm mesh sieves for microplastic sampling in wastewater reflects a compromise between methodological feasibility and ecological relevance. Smaller sieves (e.g., <20 μm) are prone to clogging from organic debris and suspended solids, hindering large-scale sampling (Murphy et al., 2016). These sieve sizes align with wastewater treatment infrastructure, which typically retain particles above 20 μm, reducing the risk of environmental release (Talvitie et al., 2017). While this approach may underrepresent microplastics <20 μm, it ensures operational efficiency and reproducibility across studies, critical for regulatory and comparative research (Koelmans et al., 2019).

- Koelmans, A.A., Nor, N.H.M., Hermsen, E., Kooi, M., Mintenig, S.M. and De France, J. (2019) ‘Microplastics in freshwaters and drinking water: Critical review and assessment of data quality’, Water Research, 155, pp. 410–422. doi:10.1016/j.watres.2019.02.054.
- Murphy, F., Ewins, C., Carbonnier, F. and Quinn, B. (2016) ‘Wastewater treatment works (WwTW) as a source of microplastics in the aquatic environment’, Environmental Science & Technology, 50(11), pp. 5800–5808. doi:10.1021/acs.est.5b05416.
- Talvitie, J., Mikola, A., Koistinen, A. and Setälä, O. (2017) ‘Solutions to microplastic pollution – Removal of microplastics from wastewater effluent with advanced wastewater treatment technologies’, Water Research, 123, pp. 401–407. doi:10.1016/j.watres.2017.07.005.
  g. ***Wastewater effluent (20 L) as well as a seawater sample (20 L) were also taken to set up a simulation of the outfall scenario. What is the rationale behind doing this simulation?***

The rationale behind using 20 L samples of wastewater effluent and seawater in the outfall simulation is to ensure sufficient capture of microplastic (MP) particles for meaningful analysis. Wastewater effluent typically contains higher concentrations of MPs compared to seawater, but MPs in both matrices are often sparse and unevenly distributed. Collecting large volumes (20 L each) increases the likelihood of retaining enough MPs for quantitative analysis, methodological consistency and statistical significance. This approach balances practical feasibility (manageable sample size) with the need to capture MPs across size classes (e.g., 20–200 µm) detected in prior wastewater studies, despite potential underrepresentation of smaller nanoplastics (Imbulana et al., 2020; Stock et al., 2019; Al-Azzawi et al., 2021).

- Al-Azzawi, M.S., Kefer, S., Weißer, J., Reichel, J., Schwaller, C., Glas, K., Knoop, O. and Drewes, J.E., 2020. Validation of sample preparation methods for microplastic analysis in wastewater matrices—reproducibility and standardization. Water, 12(9), p.2445.
- Stock, F., Kochleus, C., Bänsch-Baltruschat, B., Brennholt, N. and Reifferscheid, G., 2019. Sampling techniques and preparation methods for microplastic analyses in the aquatic environment–A review. TrAC Trends in Analytical Chemistry, 113, pp.84-92.
- Imbulana, S., Tanaka, S. and Oluwoye, I., 2024. Quantifying annual microplastic emissions of an urban catchment: surface runoff vs wastewater sources. Journal of Environmental Management, 360, p.121123.
  h. ***The microcosms consisted of actual seawater and plastic pieces obtained from the coastal municipality’s wastewater effluent. What is the amount of Water and plastic taken?***

The total volume in the Erlenmeyer flask was 250 mL. Seven different microcosms were set up so as to examine the different dilution factors with controls.

Microcosm 1, Ocean outfall near the diffuser: 250 mL Erlenmeyer flask, 150 mL seawater was added and 100 mL wastewater effluent, and the different plastic pieces were subsequently collected

Microcosm 2 simulated the mixing zone at the ocean outfall also known as the initial dilution: 250 mL Erlenmeyer flask contained 200 mL of seawater, given the vastness of the ocean, and 50 mL of wastewater effluent was well as the different plastic pieces collected.

Microcosm 3 simulated the parameter furthest away from the ocean, namely the secondary dilution parameter: The dilution factor would be the greatest at this point, thus a 250 mL Erlenmeyer flask was used that contained 225 mL saline solution and 25 mL wastewater effluent and the different plastic pieces collected.

Microcosm 4 was set up to examine the change in the microbial community on the plastics after they had been exposed to seawater without any wastewater effluent, hence simulating the furthest dilution: 250 mL Erlenmeyer flask, 250 mL of seawater was added with the different plastics collected.

Microcosm 5, view the microbial community in the seawater itself: 250 mL of Seawater was left in an Erlenmeyer flask under the same conditions.

Microcosm 6: 250 mL of Autoclaved seawater and the plastics collected to view the microbial community on the plastics itself.

Microcosm 7: The positive control consisted of 250 mL wastewater effluent and the different plastic pieces collected.

These microcosms were left for 30 days at a temperature ranging between 25°C - 27°C. The Erlenmeyer flasks were sealed with sterile cotton wool and covered with foil to eliminate the risk of contamination.

i. ***Microcosm 1 was set up to simulate the parameter at the ocean outfall near the diffuser. What are the parameters?***

The parameters simulated in Microcosm 1 near the ocean outfall diffuser include physical/hydrodynamic factors like flow rates and dilution ratios, chemical/water quality indicators such as salinity, temperature, and pollutant concentrations, biological parameters like bacterial decay rates and pathogen transport, and structural/design factors including port configuration and diffuser length. These parameters are selected to replicate real-world conditions, assess environmental impacts, and ensure compliance with water quality standards. By simulating these factors, researchers can predict how wastewater disperses and affects marine ecosystems.

Dilution Dynamics: Ratios like 3:2 or 9:1 (seawater:wastewater) replicate real-world dilution near outfalls, ensuring compliance with environmental standards. These dilutions reflect a scenario of ocean dilutions in oceanic conditions allowing researchers to assess how different dilution levels affect the distribution of bacteria.

- Chang, Y., Hu, B.X., Xu, Z., Li, X., Tong, J., Chen, L., Zhang, H., Miao, J., Liu, H. and Ma, Z., 2018. Numerical simulation of seawater intrusion to coastal aquifers and brine water/freshwater interaction in south coast of Laizhou Bay, China. Journal of contaminant hydrology, 215, pp.1-10.
  j. ***These simulations were done using the following ratios, 3:2, 4:1, 9:1 of seawater to wastewater respectively and the plastic pieces obtained from the effluent to simulate the parameters in the ocean. How are these ratios determined, and does it imitate the dilution of wastewater in the ocean?***

The parameters in the context of simulating an ocean outfall near a diffuser involve key factors such as flow rates and dilution ratios. The dilution of wastewater by seawater is a critical parameter. Dilution ratios are determined based on the flow rate of wastewater and the surrounding seawater conditions, ensuring that pollutants are sufficiently diluted to meet environmental standards. The dilution ratios (e.g., 3:2, 4:1, 9:1) are determined by considering several factors such as hydrodynamic conditions such as the ocean currents, tides, and water density that influences how wastewater mixes with seawater. The discharge characteristics of wastewater into the ocean and environmental regulations regarding the standards for effluent quality and dilution that have been set to protect marine ecosystems.

The use of these specific ratios (3:2, 4:1, 9:1) in simulations is justified because they reflect a range of dilution scenarios that can occur in different oceanic conditions. For example:

- 3:2 might represent a scenario with lower seawater flow or higher wastewater discharge.
- 4:1 could reflect typical conditions where wastewater is significantly diluted by seawater.
- 9:1 might simulate optimal dilution conditions, ensuring minimal environmental impact.

These ratios allow researchers to assess how different dilution levels affect the distribution of pollutants, including microplastics, in marine environments.

- Florida Department of Environmental Protection (FDEP) (n.d.) Ocean Outfall Study Appendices. Available at: https://floridadep.gov/sites/default/files/OOS_Appendices_0.pdf (Accessed: 10 June 2024).
- TUFLOW (2012) Outfall Water Quality Modelling. Available at: https://www.tuflow.com/media/7187/tba12_outfall-water-quality.pdf (Accessed: 10 June 2024).
  k. ***All the plastics used in this experiment were rinsed with 1 mL ddH20 thrice before adding them to the microcosms to eliminate free-living or loosely attached microorganisms on their surfaces. Won’t it give the particle results as there are chances of washing off of some the natural fauna of the ocean?***

Rinsing microplastics with ddH₂O before adding them to microcosms may remove some loosely attached microorganisms, but it is unlikely to eliminate bacteria embedded in surface crevices or biofilms (Harrison et al., 2018; Kirstein et al., 2019). This approach helps focus on persistent bacterial communities strongly associated with biofilms on microplastics. Weakly attached bacteria might not contribute as strongly to long-term contamination or biofilm-associated resistance, but they might spread more easily through the system, picking up or shedding resistance genes as they go (Oberbeckmann et al., 2015; Wright et al., 2020). By standardizing initial conditions, the study can concentrate on long-term microbial colonization, which is critical for understanding the ecological impacts of microplastics in marine environments (Amaral-Zettler et al., 2020; Rummel et al., 2017).

- Amaral-Zettler, L.A. et al. (2020) ‘Ecology of the plastisphere’, Nature Reviews Microbiology, 18(3), pp. 139–151. <https://doi.org/10.1038/s41579-019-0308-0>
- Harrison, J.P. et al. (2018) ‘Microplastic-associated biofilms: A comparison of freshwater and marine environments’, Water Research, 137, pp. 362–374. <https://doi.org/10.1016/j.watres.2018.02.039>
- Kirstein, I.V. et al. (2019) ‘Dangerous hitchhikers? Evidence for potentially pathogenic Vibrio spp. on microplastic particles’, Marine Environmental Research, 150, 104764. <https://doi.org/10.1016/j.marenvres.2019.104764>
- Oberbeckmann, S. et al. (2015) ‘Marine microplastic-associated biofilms – A review’, Environmental Chemistry, 12(5), pp. 551–562. <https://doi.org/10.1071/EN15069>
- Rummel, C.D. et al. (2017) ‘Impacts of biofilm formation on the fate and potential effects of microplastic in the aquatic environment’, Environmental Science & Technology Letters, 4(7), pp. 258–267. <https://doi.org/10.1021/acs.estlett.7b00164>
- Wright, R.J. et al. (2020) ‘Plasticizer degradation by marine bacterial isolates: A proteogenomic and metabolomic characterization’, Environmental Science & Technology, 54(4), pp. 2244–2256. <https://doi.org/10.1021/acs.est.9b05228>
  l. ***Enterobacteriaceae are a large family of Gram-negative bacteria, being the focus of this study due to their abundance in wastewater. Why does Enterobacteriaceae when literature says Pseudomonas is most abundant in wastewater?***

The focus on Enterobacteriaceae in wastewater studies, despite the abundance of Pseudomonas, is due to their significant public health and environmental implications. Enterobacteriaceae, including genera like *Escherichia coli* and *Klebsiella pneumoniae*, are common in wastewater and are key indicators of fecal contamination. They are also known for their role as reservoirs of antibiotic-resistant genes (ARGs), making them critical to monitor in wastewater effluents, which can serve as hotspots for the spread of antimicrobial resistance into the environment. (Bürgmann et al., 2018; Rizzo et al., 2013). While Pseudomonas species are abundant in wastewater and play a role in biodegradation, they are less representative of fecal pollution and public health risks compared to Enterobacteriaceae. The latter's ability to cause a wide range of infections, coupled with their prevalence in untreated and treated wastewater, highlights their importance in studies focused on microbial risks and antibiotic resistance dissemination. Thus, Enterobacteriaceae are prioritized for monitoring due to their direct relevance to human and environmental health (Zhang et al., 2009; WHO, 2017).

- Bürgmann, H., Frigon, D., Gaze, W.H., Manaia, C.M., Pruden, A., Singer, A.C., Smets, B.F. and Zhang, T. (2018). ‘Water and sanitation: An essential battlefront in the war on antimicrobial resistance’, FEMS Microbiology Ecology, 94(9), pp. 1–10.
- Rizzo, L., Manaia, C., Merlin, C., Schwartz, T., Dagot, C., Ploy, M.C., Michael, I. and Fatta-Kassinos, D. (2013). ‘Urban wastewater treatment plants as hotspots for antibiotic resistant bacteria and genes spread into the environment: A review’, Science of the Total Environment, 447, pp. 345–360.
- WHO (World Health Organization). (2017). Guidelines for safe use of wastewater, excreta and greywater: Volume 2 – Wastewater use in agriculture. Geneva: WHO Press.
- Zhang, T., Zhang, X.X. and Ye, L. (2009). ‘Plasmid metagenome reveals high levels of antibiotic resistance genes and mobile genetic elements in activated sludge’, PLoS ONE, 6(10), pp. 1–8.
  m. ***MS Excel was used for descriptive statistics throughout this study. Explain this. What descriptive statistics were used?***

MS Excel was used for descriptive statistics in this study to analyze and summarize data effectively. The descriptive statistics likely included measures such as mean, median, standard deviation, and range to provide insights into the distribution and variability of microplastic concentrations, sizes, or other parameters in wastewater and seawater samples. These statistical tools help identify trends, compare datasets, and ensure reproducibility in microplastic research by offering a clear numerical summary of the collected data.

n. ***Observations based on the SEM images highlighted that the surface of the substrates might influence biofilm attachment, as plastics with a rougher surface with deep and multiple crevices showed the most biofilm formation. Have you compared the results with the smooth surface plastic?***

The study compared biofilm formation on rough-surfaced polypropylene (PP) and smooth polyethylene (PE). While rough plastics with crevices showed significantly more biofilm formation due to increased surface area and microbial adhesion points, smooth PE substrates still exhibited detectable biofilm colonization, albeit at lower densities. SEM images of PE revealed minimal biofilm clusters, likely influenced by hydrophobicity and chemical properties rather than physical texture alone. This investigation was conducted intensively on seven different plastic types most commonly found in the environment (Rezania *et al.,* 2018). The comparative analysis of biofilm dynamics across these polymers has been submitted to a peer-reviewed journal, providing detailed insights into microbial colonization patterns and their implications for plastic degradation and environmental impact.

**Figure 7:** Comparison of a rough surface plastic (PP) and a smooth surface plastic (PE).

- Rezania, S., Park, J., Din, M.F.M., Taib, S.M., Talaiekhozani, A., Yadav, K.K. and Kamyab, H., 2018. Microplastics pollution in different aquatic environments and biota: A review of recent studies. Marine pollution bulletin, 133, pp.191-208.
  o. ***Of the 127 isolates,*** ***21 were enumerated with a view to the plastics obtained from the influent and a total of 106 isolates from the plastics in the microcosms with a view to wastewater effluent. Why is there so much difference in the isolate number? Can it be due to the dilution difference as compared to the oceans?***

The significant difference in the number of isolates (21 from influent plastics versus 106 from effluent plastics in microcosms) can be attributed to several factors. First, dilution differences and the higher number of effluent microcosms played a role, as effluent conditions simulate wastewater discharge where microplastics interact with larger seawater volumes, enhancing bacterial colonization (Pinto et al., 2021). Wastewater effluent acts as a nutrient-rich hotspot for microbial attachment, promoting biofilm formation on microplastics (Medical News Today, 2022). Additionally, while natural ocean dilution ratios are high, controlled microcosms concentrate microbial activity, intensifying colonization (Hong et al., 2023; UNEP, 2021). The greater number of effluent microcosms also increases the surface area and environmental variability, raising the likelihood of capturing diverse bacterial isolates (Wright et al., 2021). Thus, the observed disparity reflects both experimental design and environmental dynamics.

- Hong, J.K., Oh, H., Lee, T.K., Kim, S., Oh, D., Ahn, J. and Park, S., 2023. Tracking the evolution of microbial communities on microplastics through a wastewater treatment process: insight into the “Plastisphere”. Water, 15(21), p.3746.
- Medical News Today (2022) ‘Microplastic waste creates hotspots of antibiotic-resistant bacteria’. Available at: https://www.medicalnewstoday.com/articles/microplastic-waste-creates-hotspots-of-antibiotic-resistant-bacteria.
- Pinto, M. et al. (2021) ‘Microbial colonization dynamics on microplastics in wastewater environments’, Journal of Hazardous Materials, 416, 126238. Available at: <https://doi.org/10.1016/j.jhazmat.2021.126238>
- UNEP (2021) ‘Microplastics in wastewater: Towards solutions’. Available at: https://www.unep.org/news-and-stories/story/microplastics-wastewater-towards-solutions (Accessed: 11 December 2024).
- Wright, R.J. et al. (2021) ‘Microplastic surfaces facilitate bacterial aggregation and biofilm development’, Environmental Microbiology Reports, 13(3), pp. 45–59. Available at: <https://doi.org/10.1111/1758-2229.12920>
  p. ***The 16S RNA Gene for forty-eight (48) isolates were sequenced and illustrated in a phylogenetic tree (Figure 7). Why were only 48 isolates chosen for sequencing?***

Forty-eight isolates were selected for sequencing because they exhibited pathogenic characteristics, specifically the presence of the hemolysin enzyme, and possessed two or more extracellular enzymes, including proteinase, DNase, and lipase. These enzymes contribute to the isolates' virulence and ability to degrade various substrates, making them of particular interest for further genetic analysis.

q. ***The genes tested for by endpoint PCR were Intl1, FOX, and MOX. Why were these genes taken, mention their importance?***

The genes *Intl1, FOX,* and *MOX* were chosen for analysis in wastewater studies due to their critical roles in antibiotic resistance. *Intl1* serves as a marker for class 1 integrons, genetic elements that play a key role in the horizontal transfer of antibiotic resistance genes among bacteria. The *FOX* and *MOX* genes encode β-lactamase enzymes, which confer resistance to β-lactam antibiotics, a class of drugs essential for treating bacterial infections. By monitoring these genes, researchers gain valuable insights into the dynamics of antibiotic resistance within wastewater systems. This knowledge aids in understanding and mitigating the spread of antibiotic resistance, as well as improving wastewater treatment processes to reduce the dissemination of resistance genes into the environment (Stalder et al., 2012; Zhang et al., 2009; Rizzo et al., 2013).

- Rizzo, L., Manaia, C., Merlin, C., Schwartz, T., Dagot, C., Ploy, M.C., Michael, I. and Fatta-Kassinos, D. (2013). ‘Urban wastewater treatment plants as hotspots for antibiotic resistant bacteria and genes spread into the environment: A review’, Science of the Total Environment, 447, pp. 345–360.
- Stalder, T., Barraud, O., Casellas, M., Dagot, C. and Ploy, M.C. (2012). ‘Integron involvement in environmental spread of antibiotic resistance’, Frontiers in Microbiology, 3, pp. 1–14.
- Zhang, T., Zhang, X.X. and Ye, L. (2009). ‘Plasmid metagenome reveals high levels of antibiotic resistance genes and mobile genetic elements in activated sludge’, PLoS ONE, 6(10), pp. 1–8.
  r. ***The MOX gene was present in the genomes of most of the isolates. On the other hand, the FOX gene was present in nine of the 59 isolates screened. Mention the number of isolates in each section clearly, and the rationale behind taking them, as the number of isolates is not constant throughout the study?***

The rationale for selecting these isolates lies in their relevance to antibiotic resistance (AMR) studies. *MOX* and *FOX* are β-lactamase genes that confer resistance to β-lactam antibiotics, which are extensively used in clinical and environmental settings (Bush & Jacoby, 2010; Paterson & Bonomo, 2005). Screening these isolates helps assess the prevalence and distribution of such resistance genes, particularly in wastewater environments, where antibiotic-resistant bacteria (ARBs) thrive due to selective pressures from pharmaceuticals and horizontal gene transfer (Rizzo et al., 2013; Bengtsson-Palme et al., 2015). By focusing on these genes, the study aims to elucidate their role in microbial resistance dynamics and their potential public health implications, including risks of ARB dissemination into ecosystems (Berendonk et al., 2015; WHO, 2021).

- Bengtsson-Palme, J. and Larsson, D.J., 2015. Antibiotic resistance genes in the environment: prioritizing risks. Nature Reviews Microbiology, 13(6), pp.396-396.
- Berendonk, T.U. et al. (2015) ‘Tackling antibiotic resistance: The environmental framework’, Nature Reviews Microbiology, 13(5), pp. 310–317. <https://doi.org/10.1038/nrmicro3439>
- Bush, K. & Jacoby, G.A. (2010) ‘Updated functional classification of β-lactamases’, Antimicrobial Agents and Chemotherapy, 54(3), pp. 969–976. <https://doi.org/10.1128/AAC.01009-09>
- Paterson, D.L. & Bonomo, R.A. (2005) ‘Extended-spectrum β-lactamases: A clinical update’, Clinical Microbiology Reviews, 18(4), pp. 657–686. <https://doi.org/10.1128/CMR.18.4.657-686.2005>
- Rizzo, L. et al. (2013) ‘Urban wastewater treatment plants as hotspots for antibiotic resistant bacteria and genes spread into the environment’, Science of the Total Environment, 447, pp. 345–360. <https://doi.org/10.1016/j.scitotenv.2013.01.032>
- WHO (2021) Global Antimicrobial Resistance and Use Surveillance System (GLASS) Report. Geneva: World Health Organization. Available at: https://www.who.int/publications/i/item/9789240027336
  s. ***Discussion. Completely rewrite the discussion as it is more of results rather than discussion.***

The discussion has been re-written.

9. In the online submission form, you indicated that your data will be submitted to a repository upon acceptance.  We strongly recommend all authors deposit their data before acceptance, as the process can be lengthy and hold up publication timelines. Please note that, though access restrictions are acceptable now, your entire minimal dataset will need to be made freely accessible if your manuscript is accepted for publication. This policy applies to all data except where public deposition would breach compliance with the protocol approved by your research ethics board. If you are unable to adhere to our open data policy, please kindly revise your statement to explain your reasoning and we will seek the editor's input on an exemption.

The nucleotide sequences were submitted to GenBank database under the accession numbers OR178026 – OR178055. The names of the isolates with the allocated accession numbers can be found in S2 in the supporting information.
