# Supplementary material for: Marine outfall discharges contribute to coastal microplastic pollution and the spread of antimicrobial resistance
Source: PLoS One. 2025 Aug 19;20(8):e0329599. doi: 10.1371/journal.pone.0329599 (PMC12364349; doi:10.1371/journal.pone.0329599)
Supplement: Attachment — Submitted filename: Response_to_Reviewers_auresp_2.docx [file pone.0329599.s004.docx]

**Marine Outfall Discharges Contribute to Coastal Microplastic Pollution and the Spread of Antimicrobial Resistance**

*R Bhikhoo^1^, K Bezuidenhout^2^, L.G Molale-Tom^1^, C Mienie^1^, CC Bezuidenhout^1^*

*^1^Unit for Environmental Sciences and Management: Microbiology, North-West University, South Africa*

*^2^South African Research Chair: Cities, Law and Environmental Sustainability (CLES)*

*North-West University, South Africa*

1. Please review your reference list to ensure that it is complete and correct. If you have cited papers that have been retracted, please include the rationale for doing so in the manuscript text or remove these references and replace them with relevant current references. Any changes to the reference list should be mentioned in the rebuttal letter that accompanies your revised manuscript. If you need to cite a retracted article, indicate the article’s retracted status in the References list and also include a citation and full reference for the retraction notice.

We thank the reviewer for this important comment. We have carefully reviewed all 174 references in our manuscript using databases such as Retraction Watch, PubMed, and publisher platforms to verify the status of each citation. We have noticed that reference 158 was withdrawn. Changes have been made to reference 49, 125 and 129. We have also added a few references. We have also ensured that all references are complete and properly formatted according to the Vancouver style, with DOIs included where available. We appreciate the opportunity to confirm the integrity of our reference list and have taken the necessary steps to ensure accuracy and reliability in our citations.

2. (The title does not represent what the work has done.....The short title misguide the reader... I thought the paper would be about regulations and would not have experimental work....One suggestion for the title is: “ Dilution of treated sewage in ocean by outfalls negatively impact water quality by harbouring microplastics with multidrug resistant bacteria “ or something like that....

We thank the reviewer for this insightful suggestion. We have revised the title to better reflect the experimental nature and core findings of the study. The new title,
**“Marine Outfall Discharges Contribute to Coastal Microplastic Pollution and the Spread of Antimicrobial Resistance”**, clearly communicates that the paper focuses on the detection of microplastics and multidrug-resistant bacteria in marine environments impacted by wastewater discharge. We believe this revised title now accurately represents the scope and findings of the study and avoids any misinterpretation that the paper is solely policy oriented.

3. Abstract: I did not understand the last sentence in the abstract: Did you mean to say dilution of sewage in NOT and answer.....?

Thank you for pointing out the unclear wording in the last sentence of the abstract. We agree and have revised it for clarity. The intended meaning was that dilution of treated sewage in the ocean does not solve the problem of microplastic and AMR contamination. We have changed the statement to: “Our findings highlight the need for more data to challenge existing marine outfall policies and the outdated notion stating that dilution alone can solve pollution problems”.

4. Line 30, in the abstract you said that: Several isolates were resistant to carbapenems (doripenem and imipenem; 9% to 27%). But you should mention previous authors that have reported that even in secondary treated effluents (many ARB resistant to last resort antibiotics (imipinem, ertapenem, etc) were isolated , as reported by SCIENCE OF THE TOTAL ENVIRONMENT, v. 857, p. 159376-159386, 2023 ) and also from tertiary treated effluent (as Environ Sci Pollut Res 29, 36088–36099 (2022). <https://doi.org/10.1007/s11356-022-18749-3>). So, you should highlight the novelty of your work compared to previous studies.

We thank the reviewer for this valuable suggestion and for providing relevant references. We have revised the abstract taking into consideration these studies that clearly highlight the novelty of our work, which investigates the persistence of multidrug-resistant Enterobacteriaceae in biofilms on microplastics exposed to marine outfall effluent, a pathway that remains less explored.

5. In addition, abstract should contain more results of the study. Half part of the abstract is explaining regulation about outfall sewage discharge into oceans etc. you should highlight the main results obtained and how they impact and improve the current knowledge about this field (AMR spread from treated domestic wastewater). Such as the following sentence: ....our study crucially demonstrates that microplastic biofilms appear to expand this survival capacity beyond Enterobacteriaceae's known limits, creating an extended reservoir for antibiotic resistance dissemination in coastal waters.

We have revised the abstract to reduce the regulatory background and include more details of the key results, such as the species isolated, resistance rates, and the novel role of microplastic biofilms in extending the survival and spread of antimicrobial resistance. We have also incorporated the reviewer’s suggested impact statement to better highlight the contribution of this work.

6. Methods: Microcosms set -up

You should show all the different microcosms and characteristics in a Table (with the control and dilution tested and explain better the type of treated effluent (wastewater) from where you collected the microplastics to seed the microcosms.. ; it is hard to follow the rationale and all the details about the microcosms in the text (in results and discussion you explained that, but this should come on Methods. The same is valid for the genes tested : int1, MOX and FOX and why they were chosen to be tested.

How many microplastic particles did you add in each microcosm? was it fixed and equal in all conditions? 50 particles? or there was no number defined / exact?

We thank the reviewer for this insightful and constructive comment. We agree that the methodology section would benefit from greater clarity and organization.

**Microcosm Setup Description**
We have now added a comprehensive table (Table 1) summarizing all microcosm conditions, effluent dilutions, volumes and the key parameters used for each treatment. This table enhances clarity by allowing readers to easily compare experimental conditions across treatments.

**Treated Effluent Description**
A detailed description of the type of effluent used is described in the methods section under **Wastewater effluent collection.** Municipal wastewater was used that undergoes secondary treatment.

**Rationale for Gene Selection (int1, MOX, FOX)**
We have added information in the Methods section under **Molecular confirmation of identity** explaining the rationale for selecting the three genes:

- int1 was selected as a marker for anthropogenic pollution and gene transfer potential.
- MOX and FOX are β-lactamase genes known to confer resistance to cephalosporins, often associated with environmental antibiotic resistance.

These genes were chosen based on prior literature indicating their prevalence in environments impacted by wastewater and microplastics.

**Number of Microplastic Particles**
We have clarified that each microcosm received a **fixed quantity of microplastics.** The plastic pieces collected on site were divided into equal amounts and distributed between the microcosms based on type and polymers collected. This was controlled across all treatments to ensure comparability.

7. Line 504: the 16S rRNA gene....Line 513: The genes tested for by endpoint PCR were Intl1, FOX, and MOX. Did you presented in methods why you have chosen these genes and the primers used? what is FOX and MOX stands for?

Thank you for pointing this out. We agree that additional explanation is needed in the Methods section to clarify the rationale for gene selection and to specify primer sequences. We have now added:

- Information in the Methods section under **Molecular confirmation of identity** explaining the rationale for selecting the three genes
- A table (Table 2) listing the **primer sequences, annealing temperatures,** and **expected amplicon sizes** for each gene.

8. Line 570 to 572: revealed concerning multidrug resistance (MDR) patterns, with all identified species resistant to two or more antibiotics ??? did you mean to say more than 2 classes of antibiotics ? because, MDR is When the bacteria is resistant to 2 or more than 2 different antibiotic classes, according to Magiorasko. And some bacteria has intrinsic resistance to some antibiotics, did your account for that ? and considered Only acquired resistance ?

We acknowledge that the original wording was ambiguous and did not adhere strictly to the standardized definition of **multidrug resistance (MDR)** as proposed by **Magiorakos et al. (2012)**, which defines MDR as **non-susceptibility to at least one agent in ≥ three antimicrobial categories**. To address this, we have revised the sentence in the manuscript (Line 570–572) to reflect this precise definition. We also clarified whether resistance observed was potentially intrinsic or acquired by:

- **Cross-referencing the resistance profiles with established intrinsic resistance data.**
- **Reclassifying MDR only when resistance to ≥3 distinct antibiotic classes was confirmed as likely acquired**, excluding well-known intrinsic resistances.

9. Conclusions: Please be more straight forward and say the most important results, conclusions and impact of the findings of this work. Try to write the 5 most important sentences (as highlights of your study here), and does not repeat results.

We have rewritten the conclusion to be more concise and focused. The revised section now clearly presents five key highlights that reflect the most important findings and their implications, without repeating earlier results. These are:

- Microplastics serve as reservoirs for clinically relevant, multidrug-resistant bacteria in coastal waters impacted by marine outfalls.
- Marine outfall discharges, even after secondary treatment, enable the persistence and spread of antimicrobial resistance in the marine environment.
- Biofilms formed on microplastics provide a protective niche that extends the survival of Enterobacteriaceae beyond conventional expectations.
- The current regulatory framework on marine outfalls overlooks the role of microplastics in AMR dissemination and needs to be revisited.
- Our findings emphasize the urgent need for integrated monitoring of microplastics and AMR in treated effluent discharged into marine systems.
- We believe this revised conclusion provides a strong, high-impact summary of the study and its contribution to the field.
